# Psychiatrists role in primary health care in Greece: findings from a quantitative study

**DOI:** 10.1186/s13033-017-0172-0

**Published:** 2017-10-23

**Authors:** Kyriakos Souliotis, Eirini Agapidaki, Chara Tzavara, Marina Economou

**Affiliations:** 1 Faculty of Social and Political Sciences, Department of Social and Education Policy, University of Peloponnese, Damaskinou & Kolokotroni Str., 20100 Corinth, Greece; 2 Health Policy Institute, Athens, Greece; 3 Health Policy Institute, 36-38, Amaryssias Artemidos Str., 15124 Athens, Greece; 4 University Mental Health Research Institute (UMHRI), Athens, Greece; 5 First Department of Psychiatry, Medical School, University of Athens, Eginition Hospital, Soranou Ephesiou st., 115 27 Athens, Greece

**Keywords:** Mental health, Primary healthcare, Integration, Psychiatrists

## Abstract

**Background:**

Although the need for integration of mental health services into primary care is well established little has been done. The outbreak of the recession found the Greek mental health system in transition. As a response to the crisis, governments implemented horizontal budget cuts instead of health reforms. This resulted in an unfavorable situation for mental health which was set once again on the sidelines of the health policy agenda. Previous studies suggest that the most prevalent disorders in the years of financial crisis in Greece are depression and anxiety while a general increase of the psychiatric morbidity is observed does not follow the population’ needs.

**Methods:**

The present descriptive study was carried out between March and June of 2015. A convenience sample of 174 psychiatrists and psychiatry residents who met the inclusion criteria were finally selected to participate. Data were collected by using a 40-items questionnaire consisted of three sections: (a) nine questions about demographics, (b) nine questions pertaining to general aspects of administrative regulations related to primary care, (c) 22 questions about psychiatrists attitudes and perceptions towards their role in primary care. Quantitative variables are expressed as mean values, while qualitative variables as absolute and relative frequencies.

**Results:**

The vast majority of participants perceives the public primary care services and mental health services in their community as inadequate and considers psychiatrists’ participation in primary care as important in order to improve the detection and management rates of people demonstrating mental health symptoms. They also believe that: (a) primary care practitioners’ usually fail to detect the mental health conditions of patients; (b) their participation in primary care will decrease the social stigmatization for mental health conditions; (c) patients receiving pharmaceutical treatment for mental health problems by GPs and other primary care professionals usually fail to comply.

**Conclusions:**

Respondents in the present study are receptive to participate in primary care. They believe that their inclusion to primary care will result to decreased social stigmatization for mental health problems, increased patient’ access and improved detection and management rates for common mental health conditions.

## Background

The need for integration of mental health services into primary care is well established. The high prevalence and burden of mental disorders, the co-morbidity with chronic health conditions as well as the treatment gap for mental health patients, the universality of primary care, its affordability, cost-effectiveness and association to positive health outcomes substantiate the necessity and the benefits of mental health integration into primary care [1–6]. According to World Health Organization (WHO) [7], mental health services in primary care pertaining to every detectable and diagnosable mental health condition that influence physical and mental health and wellbeing. Specifically, the WHO suggests that such services include “(a) first line interventions that are provided as an integral part of general health care; and (b) mental health care that is provided by primary care workers who are skilled, able and supported to provide mental health care services” [7].

The high prevalence of mental disorders implies that there are increased needs to be addressed by effective mental health services. Regrettably, in most European countries there is a gap between the people who need help for mental health issues and those who actually receive it [8]. This treatment gap does not allow patients to have access to appropriate, adequate and effective care [9]. Evidence suggests that almost half of patients, who seek for help in the context of primary care in European countries, remain undetected and untreated [10] while the number of in-patients with mental health disorders has been increased and climbed up to 3.5 million [11, 12]. This would be attributed mainly to the failure of early detection and effective management [8]. It is estimated that 1/8 of people in general population have been hospitalized for mental health problems [12]. The low levels of identification and treatment of mental health conditions in primary care have a multilevel interpretation. Barriers exist at physician’s, patient’s and system’s level as well as in the context of health policy [7]. The key barriers at physician’s level include: lack of time, knowledge and awareness, misconceptions about mental health disorders, fear of stigmatizing patients, and limited skills [13–16]. Factors such as fear of social stigmatization, lack of awareness about mental health conditions, socio-economic obstacles and underestimation of mental health deprive the opportunity for patients to seek for help [7, 17–19]. On the other hand, the high work load which is a common aspect of many primary care systems in Europe, fragmentation of the health services, lack of cooperation between health and mental health services but also inadequate multidisciplinary collaboration between the therapeutic team members are the main systemic factors associated to the under-recognition and management of mental disorders in primary care [1, 20–22]. One of the main inhibitors at health policy level is that mental health in terms of strategic planning, public health expenditure and sustainability is not high on the agenda in many European countries [7, 22–24].

In Greece the first attempt for the re-orientation of the mental health system to a more community based approach was in late ‘90s [25, 26]. The reform was implemented in two phases: (a) Psychargos I and (b) Psychargos II programs [25]. The second phase of the Psychargos program was held from 2000 to 2010 and was focused on and targeted to the development of community mental health services in order to meet the needs of the general population and facilitate the deinstitutionalization of chronic psychiatric patients [27]. The underline principle of the reform was a shift from the patient in the psychiatric hospital to the patient in the community [28]. Primary care in Greece is provided by the public and private sector. The main services and providers involved are: (a) The outpatient clinics of the public hospitals affiliated to the public national health system; (b) Primary care units affiliated to local authorities targeted to vulnerable groups and people living below the poverty level and (c) The private clinics and practices (solo practitioners) having contractual agreements with the national insurance fund EOPYY [29]. Since a gate keeping system does not exist in Greece, patients have direct access to specialists [29, 30]. This is partially due to the lack of General Practitioners (GPs) [12]. The main role of the specialists in primary care is to address the non communicable diseases and manage chronic patients [30].

Regrettably, the second phase of the program was never completed [31]. Many community mental health services (day care centers, social care services, primary mental health services) have been developed mainly at the urban areas but the goal for an effective, comprehensive, integrated and sustainable mental health system was never reached [25, 31].

Evidence suggests that financial crisis in Greece has impacted both the mental health of the population and the mental health system [32, 33]. The outbreak of the recession found the Greek mental health system in transition [34]. As a response to the crisis, governments implemented horizontal budget cuts instead of health reforms [34]. This resulted to an unfavorable situation for mental health which was set once again on the sidelines of the health policy agenda [25, 31]. Previous studies suggest that the most prevalent disorders in the years of financial crisis in Greece are depression and anxiety while a general increase of the psychiatric morbidity is observed [35, 36]. Furthermore, it is estimated that one out of four people in Greece live in extreme poverty [37]. Vulnerable groups are affected disproportionally by social inequalities in health [38]. The limited public health expenditure for mental health, the lack of strategic planning and effective implementation of reforms combined with the increased morbidity and the limited access of people seeking help for mental health problems, have resulted in treatment gap and increased unmet mental health needs of the Greek population’ [25, 33].

The integration of mental health services into primary care would be beneficial in terms of patient access, effectiveness and sustainability of the health care system [7]. This approach is considered as a best practice not only for low and middle income but also high income countries [4]. To be effective, this strategy should be implemented by taking into account the international guidelines and best practices as well as the specific aspects, resources and features of the health system in each country [7]. For instance, Greece has the highest number of physicians per 100,000 inhabitants in EU-28 but the majority of them are specialized [12]. According to EUROSTAT [12] the total number of physicians is 68.807 while 5.322 out of them are GPs and 2.340 psychiatrists [12]. The ratio of GPs per 100,000 inhabitants is 49, the third lower in EU-28 and 21 for psychiatrists, one of the higher ratios in EU-28 respectively [12]. This implies that the number of general practitioners is not sufficient so as to staff the primary care services and deliver mental health services and programs. A potential answer to this challenge would be the inclusion of psychiatrists to primary care. Congruent with this, the present quantitative descriptive study aimed to investigate the perceptions and attitudes of psychiatrists towards their role in primary care.

## Methods

### Participants and procedures

The study was carried out between March and June of 2015. Participants were recruited from the outpatient psychiatric clinics of public general hospitals, private practices (solo practitioners) and hospitals, public health centers and community mental health centers. Since the majority of the public health services are located in the four larger urban areas of Greece: Athens, Thessaloniki, Heraklion and Patra [39], services and practices from these areas were included in the study. To be eligible for participation, individuals should be above 18 years old, understanding and speaking Greek sufficiently and be residents in psychiatry or specialized psychiatrists in one of the abovementioned cities. The Institutional Review Board of the University of Peloponnese approved the study and written informed consent was obtained by all participants. A convenience sample of 174 psychiatrists and psychiatry residents who met the inclusion criteria were finally selected to participate in the study.

Data were collected via mail by using a 40-items questionnaire of three sections: (a) nine questions about demographics, (b) nine questions pertaining to general aspects of administrative regulations related to primary care, (c) 22 questions about psychiatrists attitudes and perceptions towards their role in primary care. Close-ended questions (e.g. yes or no) and Likert type-scale items were used. The study instrument was developed according the themes and key factors emerged from an extensive literature review and considered to be essential for the integration of mental health services into primary care and the role of psychiatrists. The study questionnaire was pilot tested and revised before final administration.

### Statistical analysis

Quantitative variables are expressed as mean values (SD), while qualitative variables as absolute and relative frequencies.

## Results

The study sample was consisted of 174 physicians, 56.3% men and 43.7% women. The 83.9% of them were specialized in psychiatry. The majority (23.6%) had sixteen (16) or more years of experience and worked at a private solo-practice (56.3%). The 42.5% see ten (10) patients per month at maximum for first time psychiatric assessment while the 51.7% see fifty (50) patients per month at maximum for re-assessment and/or drug prescription (Table 1).

**Table 1:** Sample characteristics

	N (%)
Sex	
Men	98 (56.3)
Women	76 (43.7)
Age, mean (SD)	44 (8.2)
Residency	
Completed	146 (83.9)
Not completed	28 (16.1)
Work years after residency	
Less than 2	28 (19.4)
2–5	30 (20.8)
6–10	32 (22.2)
11–15	18 (12.5)
16 or more	34 (23.6)
Work site	
Public hospital	46 (26.4)
Health center	6 (3.4)
Mental health center	4 (2.3)
Private office	98 (56.3)
Private hospital	4 (2.3)
Other	16 (9.2)
Work years in current site	
Less than 1	18 (10.3)
2–3	44 (25.3)
4–10	68 (39.1)
11–15	14 (8.0)
16 or more	28 (16.1)
Monthly number of patients for first time assessment	
Less than 10	74 (42.5)
Less than 20	52 (29.9)
Less than 30	32 (18.4)
Less than 50	12 (6.9)
More	2 (1.1)
Monthly number of patients for re-assessment/drug prescription	
Less than 50	90 (51.7)
Less than 100	26 (14.9)
Less than 150	16 (9.2)
Less than 200	26 (14.9)
More	14 (8.0)
Do you have a contractual agreement with the national social insurance fund	
Yes	46 (26.4)
No	128 (73.6)

Table 2 shows the attitudes, beliefs and perceptions of psychiatrists’ about their role in primary care. The vast majority perceived the public primary care services (71.2%) and mental health services in their community (68.9%) as inadequate (agree and strongly agree) and considered their role in primary care as important in order to improve the detection and management rates of people demonstrating mental health symptoms (86.2% for agree and strongly agree). Participants also believe that primary care practitioners’ usually fail to detect the mental health conditions of patients (64.4% for agree and strongly agree).

**Table 2:** Attitudes about the perceived role of psychiatrists in primary care

	Do not know/do not answer	Strongly disagree	Disagree	Slightly disagree	Slightly agree	Agree	Strongly agree
	Ν (%)	Ν (%)	Ν (%)	Ν (%)	Ν (%)	Ν (%)	Ν (%)
The public primary care services in my community are inadequate	0 (0.0)	0 (0.0)	4 (2.3)	16 (9.2)	30 (17.2)	50 (28.7)	74 (42.5)
The public mental health services in my community are inadequate	0 (0.0)	0 (0.0)	10 (5.7)	22 (12.6)	22 (12.6)	54 (31.0)	66 (37.9)
I believe that psychiatrist’ participation in primary care is necessary in order to ensure a holistic approach to health of patients	0 (0.0)	0 (0.0)	0 (0.0)	4 (2.3)	16 (9.2)	40 (23.0)	114 (65.5)
People prefer to seek help for mental health problems in primary care to avoid the social stigmatization that accompanied mental health services	4 (2.3)	8 (4.6)	10 (5.7)	16 (9.2)	38 (21.8)	62 (35.6)	36 (20.7)
I believe that psychiatrist’ participation in primary care will significantly improve the detection and management rates of people demonstrating mental health symptoms	0 (0.0)	0 (0.0)	4 (2.3)	4 (2.3)	16 (9.2)	42 (24.1)	108 (62.1)
I believe that the primary care practitioners and physicians do not realize the necessity of psychiatrists’ participation in primary care	4 (2.3)	0 (0.0)	20 (11.5)	24 (13.8)	36 (20.7)	30 (17.2)	60 (34.5)
Primary care practitioners’ usually fail to detect the mental health conditions of patients	0 (0.0)	0 (0.0)	4 (2.3)	14 (8)	44 (25.3)	48 (27.6)	64 (36.8)
Patients’ receiving pharmaceutical treatment for mental health problems by GPs and other primary care professionals usually fail to comply	0 (0.0)	0 (0.0)	6 (3.4)	4 (2.3)	20 (11.5)	72 (41.4)	72 (41.4)
The improvement of health care delivery for patients having mental health problems is strongly related to the collaboration between the psychiatrist and the primary care team members	6 (3.4)	0 (0.0)	2 (1.1)	0 (0.0)	4 (2.3)	62 (35.6)	100 (57.5)
GPs and psychiatrists are equally effective in the use and management of psychiatric drugs	0 (0.0)	96 (55.2)	60 (34.5)	4 (2.3)	10 (5.7)	2 (1.1)	2 (1.1)
The management of mental health conditions in primary care by psychiatrists may significantly contribute to the decrease of social stigmatization	0 (0.0)	0 (0.0)	4 (2.3)	2 (1.1)	20 (11.5)	86 (49.4)	62 (35.6)
The mental health services delivery exclusively by mental health specialists, may significantly contribute to the increase of social stigmatization for mental health conditions	0 (0.0)	54 (31)	92 (52.9)	20 (11.5)	4 (2.3)	2 (1.1)	2 (1.1)

Respondents’ perceptions about patient access to mental health services can be found at Table 3. The vast majority believe that patients with mental health problems do not have adequate access to psychotherapy (95.4% for agree and strongly agree) while 75.9% of those attributed the limited access to out of pocket cost. Furthermore, the 71.3% (for agree and strongly agree) of the participants believe that patients do not have adequate access to pharmaceutical treatment while the 72% ascribed it to the patients inability to pay and/or the limited health insurance coverage. They suggest that treatment costs (for psychotherapy and pharmaceutical treatment) should be covered by the public national health insurance fund.

**Table 3:** Opinions about access to mental health services

	Ν (%)
Do you think that patients with mental health problems have adequate access to psychotherapy?	
Yes	8 (4.6)
No	166 (95.4)
If not, why?	
Because the public national health insurance do not cover psychotherapy costs and patients cannot afford it	126 (75.9)
Due to the lack of trained psychiatrists to psychotherapy techniques and models	20 (12.0)
Because patients are not receptive to psychotherapy	4 (2.4)
Other	16 (9.6)
Do you think that patients with mental health problems have adequate access to pharmaceutical treatment?	
Yes	124 (71.3)
No	50 (28.7)
If not, why?	
Because many patients do not have health insurance coverage and cannot afford the out of pocket treatment cost	36 (72.0)
Due to bureaucratic barriers in prescription execution	0 (0.0)
Due to the lack of availability of psychiatric drugs in pharmacies	2 (4.0)
Because patients are not receptive to take pharmaceutical treatment for mental health problems	10 (20.0)
Other	2 (4.0)
The public national health insurance fund should cover	
The total cost of psychotherapy treatment for a certain period of time	42 (24.1)
Part of the cost for psychotherapy treatment for a certain period of time	60 (34.5)
The total cost of psychotherapy treatment for the time needed, without any restrictions	64 (36.8)
The public national health insurance fund should not cover psychotherapy costs	0 (0.0)
Do not know/do not answer	8 (4.6)

## Discussion

The present study aimed to investigate psychiatrists’ attitudes and perceptions about their involvement in primary care. Our results are in line with previous efforts in the field [40–44] and indicated that psychiatrists and psychiatric residents are receptive to participate in primary care although important barriers were also cited. Norfleet et al. [41] surveyed fifty-two psychiatrists working in an integrated primary care model in an effort to describe their role and elicit their views and opinions. They found that they were very satisfied with their experience in primary care and the communication and collaboration with primary care professionals. The effective teamwork was highlighted as a key element of the model. McGrath [42] explored the perspectives of primary care practitioners and psychiatrists about their role and responsibilities in primary care. Study results indicated that primary care practitioners were more confident dealing with mental health problems than psychiatrists felt in treating physical conditions. As regards the main barriers to effective communication, psychiatrists cited time constraints and primary care practitioners confirmed this inhibitor by underlying the unavailability of psychiatrists. It seems that the interdisciplinary teamwork is a challenging process requiring training and the development of a collaborative relationship among the professionals of different disciplines and specialties. Raney [43] emphasized the value of comprehensive and holistic care in the context of a collaborative model in primary care. She suggested that psychiatrists should be able to manage the physical problems of their patients and primary care practitioners should be able to address the mental health conditions of patients suffering physical chronic conditions. Moreover, Jones et al. [44] found that primary care practitioners and psychiatrists hold slightly negative attitudes towards older people with schizophrenia. The provision of care to this patient group was rated as inadequate but it was attributed to the inefficient communication, time constraints and ineffective collaboration between practitioners of different specialties (primary care physicians and psychiatrists). On the other hand, Golomb et al. [40] in their study used a multidisciplinary panel to assess whether psychiatrists in primary care are able to provide prevention and treatment for physical conditions. Their results revealed that primary care practitioners and administrators believe that psychiatrists working in primary care have the skills to provide such services to patients suffering from mental health conditions.

Psychiatrists in the present study perceive the clinical mental health practice of primary care practitioners as well as mental health services as inadequate. A recent study in Greece investigated the role of pediatric primary care providers in detection and management of maternal mental health problems [45]. It was found that the lack availability of mental health services and professionals as well as the lack of free-accessed community mental health services, the fragmentation of primary care, the ineffective communication and collaboration between primary care and mental health services and professionals and the inadequate continuity of care were the main inhibitors for the detection and management of maternal mental health problems in the context of primary care [45]. Other studies in Greece have also identified the multiple organizational deficiencies of the mental health services [25, 46]. The most common are: lack of strategic planning, implementation and evaluation of a sustainable mental health system, unequal geographical distribution of mental health services between urban and rural areas as well as unequal distribution of the services provided in different mental health centers located in the same area, lack of mental health services for vulnerable groups (e.g. children, elderly), limited intersectoral collaboration, lack of monitoring and quality assurance methods and tools, defective governance [25, 46]. Although Greece has high public health expenditure, the mental health budget remains low and access to mental health services is mainly depended on out of pocket payments. As a result, the high public health expenditure cannot be translated into improved mental health outcomes [46].

Respondents in our study also highlighted the limited access of primary care patients to psychotherapeutic and pharmacological treatment. It was suggested that treatment costs should be covered by the public health expenditure in order to facilitate the patients’ access to appropriate and effective mental health care. In Greece psychotherapy is not covered by the national health insurance [47]. Free accessed—or at low cost—psychotherapy is provided by few non-governmental organizations and the community mental health centers which are scarce and located in large urban areas [47–50]. The majority of patients seek for help at the private sector due to the long waiting lists and the lack of continuity of treatment [27, 49]. Moreover, patients have to pay an important amount of money (there is a 25% average copayment rate for each prescription) for pharmaceutical treatment [49, 50]. Only patients living in extreme poverty and have a valid social welfare insurance coverage may have free of charge access to pharmaceutical treatment [15]. It should be noted that since most mental health professionals are located in urban areas, the financial burden for patients is further increased due to transportation costs [35].

Physicians in our study reported that the inclusion of psychiatrists in primary care will decrease the social stigmatization for mental health problems, increase population access to qualitative mental health services and improve the detection and management rates of patients with mental disorders. Evidence suggest that the provision of mental health services in primary care has been associated with decreased social stigmatization [3, 7, 20, 51–53]. The long term relationship developed with the primary care physician facilitates the disclosure of mental health concerns [54] Congruent with this, the integration of mental health services in primary care and the development of collaborative care models are part of the best practices to decrease stigma and improve the mental health outcomes of the population [7, 20, 26]. Goodrich et al. [20] advocated that stepped collaborative care models should be implemented to decrease stigmatization and not just the co-location of mental health specialists into primary care. Butler et al. [52] also suggested that mental health services should be delivered in the primary care in order to address stigmatization for mental health conditions and they highlight the importance of onsite provision of such services from a patient perspective. Of course, the integration of mental health services in primary care itself is a necessary but not sufficient condition to reduce stigmatization for mental health problems. Multifaceted community interventions are also needed to address misconceptions and unfavorable attitudes about mental health and improve the social support of patients suffering from psychiatric disorders [7].

Over the last few years more sophisticated models and approaches have been developed for the integration of mental health services into primary care [5, 55]. Specialized physicians are not included in most programs and strategies. On the contrary they invest on adequately trained primary care professionals such as GPs for the provision of mental health services but both approaches stem from the chronic care model and the collaborative care [56, 57]. The WHO [7] emphasizes that adequate training and support should be provided to primary care professionals in order to address effectively the mental health needs of the population but underlines that there is no a “single best practice model” for the integration. Moreover, it suggests that each country should endorse and adapt best, good and promising practices by taking into account the specific aspects and resources of the health system [7]. Although there are different approaches for the integration of mental health into primary care, however fundamental principles do also exist and prescribe that primary care should be provided by a multidisciplinary team, an effective coordination and collaboration between health and mental health services should be developed and sustainable resources must be reserved in order to ensure the continuity of care [58–62].

The outbreak of the financial crisis in Greece found the health system in transition [34]. The weaknesses of the system have been increased due to the political choice of horizontal budget cuts as a response to the recession and the government’s hesitation to design, implement and evaluate a coherent, concrete and sustainable plan of health reforms [34, 48]. As a result, the health system is characterized by ineffectiveness, mental health was set aside in health policy agenda while the mental health problems continue to rise [25, 31, 34, 48]. The high unemployment rates together with the social inequalities in health and the unmet needs of the population shape the main challenges that should be targeted [36, 38].

The integration of mental health into primary care in Greece should be a key priority for Greece and must take into account the strengths and weaknesses of the health system as well as the availability of resources. Since the number of GPs in Greece is under the European average [12], the majority of public health services are located in urban areas [39] and the recession continues to impact health and healthcare [25], the inclusion of psychiatrists in primary care could be a potential solution for the expansion of services so as to meet the mental health needs of the population. Of course, the participation of specialized physicians in primary care is deemed as a choice of high cost for the national health system and one may argue that implies limited access for patients [63]. Since primary care services in Greece are provided by both the public and private sector, perhaps psychiatrists negativity of the primary care practitioners capacity to manage mental health conditions can be understood on the basis that they are market competitors. As stated above, patients in Greece seek for mental health services mainly in the private sector. If the responsibility of managing mental health problems is shared among physicians of different specialties in a context of public–private partnership (e.g. by having contractual agreements with the national insurance fund) then the reimbursement will be also shared. Studies contrasting the views of psychiatrists and primary care practitioners about their role in primary care provide some insights on the issue. Although psychiatrists in Sun et al. [54] study hold more favorable attitudes about primary care professionals capability to provide mental health services compared to our results, however they did not support primary care physicians in this role. Primary care practitioners cited lack of collaboration and communication with psychiatrists as key barriers for the effective mental health management in primary care [54]. In a French study, psychiatrists from the private sector doubted the capability of primary care practitioners to manage mental health conditions while their colleagues of the public sector hold opposite opinions and believe that primary care professionals are adequate in addressing such conditions [64]. This may reflect the assumption of market competitors that was also suggested for the interpretation of our results. On the contrary, general practitioners and psychiatrists in Lucena et al. [65] study were not receptive to the integration of mental health services in primary care and the co-location—or periodically visits- of psychiatrists in primary care.

The out of pocket costs for mental health services in Greece remain high notwithstanding the financial crisis [66]. This is due to the ineffectiveness of the public health services, the long waiting lists, and the limited access to specialized care for the unemployed [66, 67]. Contractual agreements have been arranged between the public and private sector for employed people who have national public health insurance in an effort to address the unmet physical needs of the population [29, 67]. In this case, the cost is covered by the national public health insurance fund [29]. This strategy could be implemented for the case of mental health as well. Unemployed individuals will have access to trained GPs and psychiatrists in public primary care services and employed patients may have access to psychiatrists in the private sector. In addition, psychiatrists from the private sector may also provide services to rural and semi-urban areas, where public primary care services are scarce [29, 39].

Findings in the present study are subjected to the following limitations. The use of convenience sampling implies that the results cannot be generalized to the total population of psychiatrists. Since the large number of public health services is located in urban areas, respondents recruited only from those units. Perhaps psychiatrists resident in semi-urban and rural areas would reveal different views and opinions towards their role in primary care. Moreover, this is a descriptive study in an under-investigated issue in Greece. We cannot assume that there are associations between psychiatrists’ views and perceptions formulating a hypothesis about the different aspects of their role in primary care.

## Conclusions

The high prevalence and burden of mental disorders, the co-morbidity with chronic health conditions such as non-communicable diseases as well as the treatment gap for mental health patients, the universality of primary care, its affordability, cost-effectiveness and association to positive health outcomes substantiate the necessity and the benefits of mental health integration into primary care [7, 8]. Our findings are congruent with previous efforts in the field [40–44] indicating that psychiatrists and psychiatric residents in our sample are receptive to participate in primary care. A future comparative study including psychiatrists and primary care practitioners would be more informative about the key aspects of the integration of mental health services into primary care. Moreover, the views and perceptions of patients, general population groups and policy makers are of great importance in order to develop a deeper understanding about the different aspects of the integration of mental health services into primary care and develop plans and reforms according to the needs, assets and weaknesses of the stakeholders.
